# Macon Medical Association

**Published:** 1871-05

**Authors:** 


### MACON MEDICAL ASSOCIATION.

MACON, GA., May 2, 1871.

The Association met and was called to order by its President, Dr. Wm. F. Holt. A full meeting was present. It being the anniversary of this Association, the regular business was suspended for the purpose of hearing the report of Dr. Nottingham, our representative to the State Medical Association at Americus. In obedience to this provision, the Doctor proceeded with a most minute and accurate report of the proceedings, &c. At its conclusion, a committee of three was on motion appointed to take such steps as in their judgment, the late action of the Georgia Medical Association demands. The committee, in due time presented the following preamble and resolutions, which were unanimously adopted:

*Whereas*, the recent meeting of the Georgia Medical Association, held in the city of Americus, of which body we in our individual capacity are permanent members, and at which meeting we as a Society had a representative, did see fit and proper to go back on the record for three years, and rescind by resolution all the action of the Association at the meetings in Augusta in 1868, in Savannah in 1869, and in Macon in 1870, in regard to the Faculty (of 1868) of the Atlanta Medical College; and

*Whereas*, however unpleasant and painful to us as individuals may have been the action of the Association at these several meetings in reference to the persons composing said Faculty, particularly that taken at Macon in 1870, still we have always felt that it was in the main justifiable and proper; therefore be it

1. *Resolved*, That the action taken at Augusta, in 1868, by the Association, in its resolution of unwillingness to recognize the prospective graduates of the Atlanta Medical College, was just and proper, in view of the great abuses in the matter of graduation, to which the amended charter of said College invited its Faculty.

2. *Resolved*, That the resolution passed at the meeting in Savannah in 1869, declaring that the self-respect of the Association required, notwithstanding the disclaimer already received, that the Faculty of the Atlanta Medical College (of 1868) should make, *in print*, a withdrawal of the offensive terms and language contained in a memorial addressed by them in pamphlet form, to the Legislature of the State, *was not unreasonable, and should in our judgement have been promptly complied with by said Faculty.*

3. *Resolved*, That the failure of the Faculty to make the *amende honorable* in the manner in which they had made their charges, and in the manner required by the resolution of the

Association at Savannah, was sufficient cause for their expulsion when it became known that twelve months had elapsed in which they could have done so.

4. *Resolved*, That in the recent action at Americus, in reference to these persons, we believe a great wrong has been done the Association—particularly those members of it who composed the meeting at Augusta in 1868, denounced in the celebrated memorial—and that we fully, unequivocally and cordially endorse the protest against said action at Americus, spread on the minutes of the meeting, and signed by thirteen members, our representative among the number.

5. *Resolved*, That the opinion which we learn was entertained and expressed by gentlemen from Atlanta, that the physicians of Macon kept away from the Americus meeting because of the action taken in their city last year, is without a shadow of foundation in fact or truth, and that we take this occasion to say two Macon physicians, members of this Society, were at Americus in the interest of peace, harmony and justice, and we wish we all could have been there ; and further, that whilst the expulsion of the members of the Atlanta College Faculty (of 1868) was painful to us, we did not then perceive, nor do we now see, that there was any other alternative, except so far as Dr. H. V. M. Miller is concerned, who we have very lately learned was not a signer of the memorial, and who in our opinion should not have had his name stricken from the roll.

6. *Resolvcd*, That we are pleased to hear of the action taken by the Georgia Medical Society of Savannah, in their resolutions forwarded to this body, and that we will be glad to co-operate with that old and intelligent society and with all other correct-thinking members of the Georgia Medical Association, in the adoption of such measures condemnatory and reparative of the *Logan Preamble and Resolution*, as deliberation and a free interchange of opinion may suggest as wise, prudent aad proper.

7. *Resolved*, That a committee of three be appointed to communicate these resolutions to the Medical Society of Georgia at Savanaah, and to conduct correspondence on this subject on behalf of this Association with that society, and with other aggrieved parties throughout the State.

In obedience to the last resolution, the following gentlemen were appointed corresponding committee: Dr. Wm. R. Burgess, Dr. P. H. Wright, and Dr. C. H. Hall.

The following resolution by Dr. Burgess was unanimously adopted:

" *Resolved*, That the thanks of the Macon Medical Association are due, and the same are hereby cordially tendered, to Dr. C.

B. Nottingham, our representative in the Medical Association of Georgia, at Americus, for his very minute aad accurate report of the proceedings of said Society, but *more especially for his earnest, manly and noble defence of professional right and professional honor* before the Association.

Dr. Nottingham was made next President of the Macon Medical Association.

